# Shortcomings of SARS-CoV-2 genomic metadata

**DOI:** 10.1186/s13104-021-05605-9

**Published:** 2021-05-17

**Authors:** Landen Gozashti, Russell Corbett-Detig

**Affiliations:** 1 Department of Organismic and Evolutionary Biology and Museum of Comparative Zoology, Harvard University, Cambridge, MA 02138 USA; 2 Department of Biomolecular Engineering and Genomics Institute, University of California Santa Cruz, Santa Cruz, CA 95064 USA

**Keywords:** SARS-CoV-2, Metadata, Genomics, Databases, Data quality, COVID-19

## Abstract

**Objective:**

The SARS-CoV-2 pandemic has prompted one of the most extensive and expeditious genomic sequencing efforts in history. Each viral genome is accompanied by a set of metadata which supplies important information such as the geographic origin of the sample, age of the host, and the lab at which the sample was sequenced, and is integral to epidemiological efforts and public health direction. Here, we interrogate some shortcomings of metadata within the GISAID database to raise awareness of common errors and inconsistencies that may affect data-driven analyses and provide possible avenues for resolutions.

**Results:**

Our analysis reveals a startling prevalence of spelling errors and inconsistent naming conventions, which together occur in an estimated ~ 9.8% and ~ 11.6% of “originating lab” and “submitting lab” GISAID metadata entries respectively. We also find numerous ambiguous entries which provide very little information about the actual source of a sample and could easily associate with multiple sources worldwide. Importantly, all of these issues can impair the ability and accuracy of association studies by deceptively causing a group of samples to identify with multiple sources when they truly all identify with one source, or vice versa.

## Introduction

Metadata, or “data about data,” [1] is an essential component of science: informing both data-driven analyses and decisions with regards to public health [2–6]. Consequently, inadequate metadata quality can inhibit the discoverability of relevant data and hinder epidemiological research efforts and the development of clinical policy [3, 7, 8]. In spite of this, metadata standards are sometimes neglected, and databases critical to public health related research efforts including Dryad, Genbank, BioSample (managed by the National Center for Biotechnology Information), BioSamples (managed by the European Bioinformatics Institute), the Electronic Health Record (EHR) and various other repositories are plagued by inconsistencies and erroneous metadata entries [9–18].

As some groups have previously mentioned, the severe acute respiratory syndrome coronavirus 2 (SARS-CoV-2) pandemic has shed light on metadata inadequacies, which have inhibited studies relevant to both epidemiology and viral population dynamics [18–21]. Databases such as the global initiative on sharing avian influenza data (GISAID) [22] and Nextstrain [23] have empowered an impressive array of SARS-CoV-2 studies by maintaining SARS-CoV-2 genomic sequences and corresponding metadata. GISAID is perhaps the most important database for research efforts related to SARS-CoV-2 because it is the largest and most widely used database of SARS-CoV-2 genomic variation, maintaining 223,024 SARS-CoV-2 genomic sequences as of November 27th 2020. GISAID’s purpose is to facilitate sharing of viral genome sequences and related clinical and epidemiological metadata to help researchers understand how viruses evolve and spread during epidemics and pandemics [22]. Two of these metadata categories, “originating lab” (the lab in which the sample was collected) and “submitting lab” (the lab that submitted the viral genome), are important for finding erroneous variants in SARS-CoV-2 genomes [20, 24, 25]. Here, we specifically highlight inconsistencies and erroneous entries in “originating lab” and “submitting lab” descriptions within GISAID to exemplify where improvements in metadata quality are needed and to raise awareness to data submitters and maintainers alike. Similar concerns likely affect other databases as well and we do not intend this as a criticism of GISAID; rather this is an opportunity for improvement of metadata across all databases.

## Main text

### Methodology

We initially used a previously developed method described in [20] to systematically detect cases of inconsistencies throughout GISAID’s “originating lab” and “submitting lab” metadata categories. Then we manually parsed the metadata to confirm our results and detect cases missed by the systematic method. We note that our results likely represent an underestimate of the true number of metadata inconsistencies since some cases are too divergent to resolve.

### Results and discussion

**Fig. 1:**
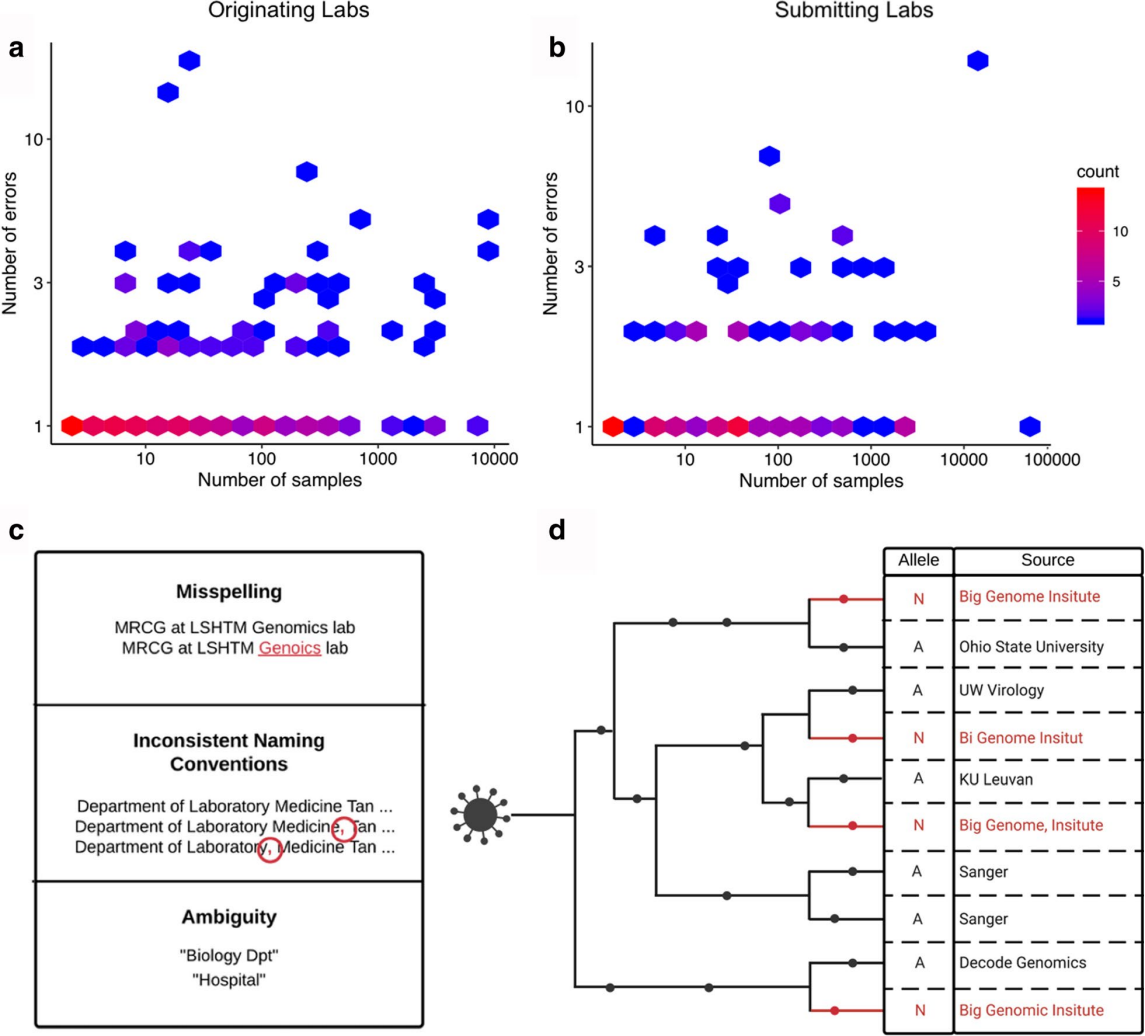
The number of samples produced by each (**a**) “originating lab” and (**b**) “submitting lab” and the corresponding number of errors (or inconsistencies) for that respective lab. Color encodes the respective number of data points at a given position on the plot, with positions with fewer points shaded blue and positions with more points shaded red. **c** Some observed examples of misspellings, inconsistent naming conventions, and highly ambiguous entries. **d** A hypothetical phylogenetic tree displaying an example of a case in which errors in “originating lab” metadata might impede association studies with regard to SARS-CoV-2 genomic data. We denote true mutations with black dots and ambiguous mutations with red dots on the phylogeny. In this case, ambiguous “N” alleles occur multiple times across a phylogeny at a given site and all stem from the same lab. Metadata errors (shown in red) cause this ambiguous “N” allele to appear as if it is associated with 4 different labs (rather than 1). Such a site could impair phylogenetic inference and should be flagged in the SARS-CoV-2 masking recommendations but could be overlooked as a result of these errors [20, 24]

We used a combination of systematic and manual approaches to estimate the prevalence of spelling errors and naming inconsistencies in “originating lab” and “submitting lab” metadata categories for all GISAID SARS-CoV-2 sequences as of November 27th 2020. We note that the fact that GISAID requires extensive metadata for each submitted SARS-CoV-2 genome is extremely valuable and represents an exemplary model for other databases for genomic data of epidemiological value. However, our analysis reveals that an alarmingly large proportion of lab names are misspelled or exhibit inconsistent naming conventions among samples at least once: ~ 9.8% and ~ 11.6% for “originating lab” and “submitting lab” entries respectively. Furthermore, we observe instances in which lab names are misspelled or named inconsistently multiple times across samples, and cases of highly ambiguous lab names such as “Hospital” or “Biology Dpt” that could be associated with multiple labs (Fig. 1a–c).

One of the primary consequences of spelling errors and inconsistent naming conventions in these particular categories (and more generally) is the appearance that a group of samples identifies with multiple labs, when they all truly identify with one particular lab (Fig. 1d). The opposite effect, where samples from disparate labs are erroneously associated with the same lab, is also possible. Both of these effects can impair association studies. Notably, “originating lab” and “submitting lab” metadata categories are pertinent to the ability to accurately identify systematic sequencing errors associated with specific sequencing groups in SARS-CoV-2 genomes and the sources and causes of erroneous variants in SARS-CoV-2 genomic data [20, 24]. The challenges with accurate interpretation of these metadata fields has led to onerous workarounds such as using “country” as an imprecise proxy for the likely origin of a sequence [25]. Concerningly, the same metadata errors we describe have been propagated into downstream analysis platforms (e.g. [26]), further highlighting a need for improved metadata quality.

There are three possible solutions to the challenges of inconsistent and inaccurate metadata. First, we urge producers of SARS-CoV-2 genomic data to proceed with caution when submitting their metadata, and advocate that maintainers of genomic databases be aware of possible errors in incoming metadata (such as those we show) and attentively promote metadata standardization. A second solution is to completely ignore samples with suspected corresponding metadata errors [18]. However, this solution can result in a significant decrease in sample size, limiting the power of statistical analyses [18]. On another hand, the development of new reliable methods for metadata correction could serve as an alternative and could likely be applied across multiple disciplines [1, 27, 28]. Methods for metadata quality evaluation and subsequent correction are in active development [4, 16, 28]. However, automated metadata correction is a nontrivial task, and future work is required to evaluate current algorithms for metadata correction and the feasibility of their application to large genomic databases like GISAID.

### Conclusion

The SARS-CoV-2 pandemic has prompted an unprecedented response from the scientific and public health community, and the development and maintenance of databases such as GISAID have permitted epidemiological and comparative studies of unparalleled power. Indeed, the size and relative uniformity of the GISAID database is the very reason this analysis is possible. However, a brief analysis reveals that the quality of metadata accompanying such datasets is sometimes unreliable. A study conducted by McMahon and Denaxas in 2016 concluded that “one of the main challenges in assessing quality in epidemiological and public health research is a lack of awareness of the issue of poor quality metadata” [4]. The SARS-CoV-2 pandemic is an unfortunate source of enlightenment to metadata shortcomings. Here we primarily focus on errors and inconsistencies, but metadata completeness and detail are of equivalent importance [21]. The importance of quality metadata with regard to our ability as a species to combat this pandemic and future pandemics is now more important than ever.

## Limitations

This work primarily focuses on issues within the GISAID database and does not consider other SARS-CoV-2 genomic databases. Thus, the extent of errors we describe throughout SARS-CoV-2 metadata in other databases remains unknown, but similar effects are likely present in other databases as well. It is also possible that GISAID exemplifies an extreme case of metadata inconsistencies and that our observations are less prevalent across SARS-CoV-2 metadata as a whole.

## Data Availability

The datasets analyzed during the current study are available in the GISAID repository, https://www.gisaid.org/.

## Abbreviations

SARS-CoV-2: Severe acute respiratory syndrome coronavirus 2
GISAID: Global initiative on sharing avian influenza data
